# Atypical Cerebral Carcinoids Treated with Hippocampal-Sparing Whole Brain Radiotherapy

**DOI:** 10.7759/cureus.74997

**Published:** 2024-12-02

**Authors:** James A Knight, Waleed F Mourad, Hafsa Nebbache, Aradhana Kaushal

**Affiliations:** 1 Radiation Medicine, University of Kentucky, Lexington, USA; 2 Radiation Oncology, University of Kentucky, Lexington, USA; 3 Pathology, University of Kentucky, Lexington, USA

**Keywords:** atypical carcinoid, brain met, hippocampal sparing, image guided radiotherapy, metastatic neuroendocrine carcinoma, whole-brain radiotherapy

## Abstract

Central nervous system (CNS) metastases of atypical carcinoid tumors are exceptionally rare. Isolated studies suggest a survival benefit in patients who receive whole-brain radiotherapy (WBRT); however, it has been known to have detrimental effects on long-term memory and executive function. Here, we present a case of a patient initially diagnosed with stage IIB bronchopulmonary carcinoid who later developed hepatic and intracranial metastases despite receiving adjuvant systemic therapy over a two-year period. She underwent hippocampal-sparing WBRT (HS-WBRT), receiving 30 Gy in 10 fractions via daily image-guided photon therapy using two coplanar arcs. Subsequent clinical evaluations and magnetic resonance imaging (MRI) of the brain at 11 months post-treatment demonstrated a decrease in the size and number of brain metastases, with the patient reporting stable memory and cognition. This case demonstrates the efficacious delivery of palliative HS-WBRT in a patient with a rare presentation of brain-metastatic atypical carcinoid, conferring effective local control and preservation of cognition. Multi-therapy regimens that incorporate HS-WBRT may be considered for improved disease control and quality of life. Further investigation of systemic agents, and possible molecular targets that could confer greater efficacy against treatment-progressive atypical carcinoids, is also warranted.

## Introduction

Carcinoid tumors are a rare, heterogeneous group of neoplasms that originate from endodermal precursor cells. They follow an indolent course and possess the capacity to produce and secrete hormones and neuropeptides, most notably serotonin (5-hydroxytryptamine or 5-HT). These tumors most commonly arise from the gastrointestinal tract (54.5%) and bronchopulmonary system (30.1%) [1]. Atypical carcinoid is a rare subtype of lung tumor that comprises 10% of pulmonary carcinoids and less than 1% of pulmonary malignancies [2]. Unlike typical carcinoids, atypical ones have an aggressive clinical course, with a relatively high incidence of mediastinal lymph node metastases and a five-year survival rate of only 40-75% [3]. Metastases in patients with carcinoids have been reported to range from 15 to 40% [4], with the most common sites being regional lymph nodes, liver, lungs, and bones [4-6]. Central nervous system (CNS) metastases are exceptionally rare, occurring in approximately 1.5% of patients with carcinoid tumors [6,7]. Uniform guidelines and literature for their management are sparse, though isolated studies suggest a survival benefit in patients who receive whole-brain radiotherapy (WBRT) [6]. Herein, we present a case of brain-metastatic atypical bronchopulmonary carcinoid, successfully treated with hippocampal-sparing (HS) WBRT and demonstrate the feasibility and safety of this treatment modality.

## Case presentation

**Figure 1:**
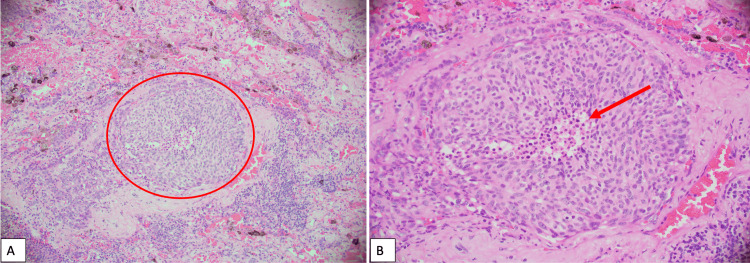
Pulmonary resection of the atypical carcinoid A) Tumor nests (circled in red) with B) central necrosis (red arrow) and “salt and pepper” chromatin.

A 62-year-old female patient with Eastern Cooperative Oncology Group performance status (ECOG-PS) of zero, and a remote history of stage IIA bulky mediastinal nodular sclerosis Hodgkin’s disease successfully treated with 35 Gy in 20 fractions, presented for surveillance scans. Computed tomography (CT) of the chest revealed a 1.0 cm left upper lobe nodule, and CT-guided biopsy revealed a bronchopulmonary carcinoid. The patient underwent video-assisted thoracoscopic surgery (VATS) left upper lobectomy with mini thoracotomy. The presence of tumor nests with central necrosis and salt-and-pepper chromatin on surgical pathology confirmed the diagnosis of atypical carcinoid tumor (Figure 1). Lymphovascular invasion and visceral pleural involvement were also identified.

The patient was subsequently classified with stage IIB (pT3N0M0) disease and was given four cycles of adjuvant cisplatin and etoposide. Two years after completing systemic therapy, the patient underwent a CT scan of the abdomen and pelvis, which demonstrated 1.3 cm, 2.4 cm, and 1.6 cm lesions within the right hepatic lobe and a 1.1 cm lesion in the left hepatic lobe. She was asymptomatic and reported no new concerns when these surveillance scans were performed. CT-guided biopsy confirmed the presence of liver-metastatic atypical carcinoid and systemic therapy with capecitabine and temozolomide was initiated. Due to the presence of these liver metastases, gallium-68 dotatate positron emission tomography-computed tomography (^68^Ga-PET/CT) and contrasted magnetic resonance imaging (MRI) of the brain were performed shortly after biopsy to survey the extent of metastatic disease. MRI brain revealed multiple punctate contrast-enhancing lesions in the supratentorial and infratentorial brain.

**Figure 2:**
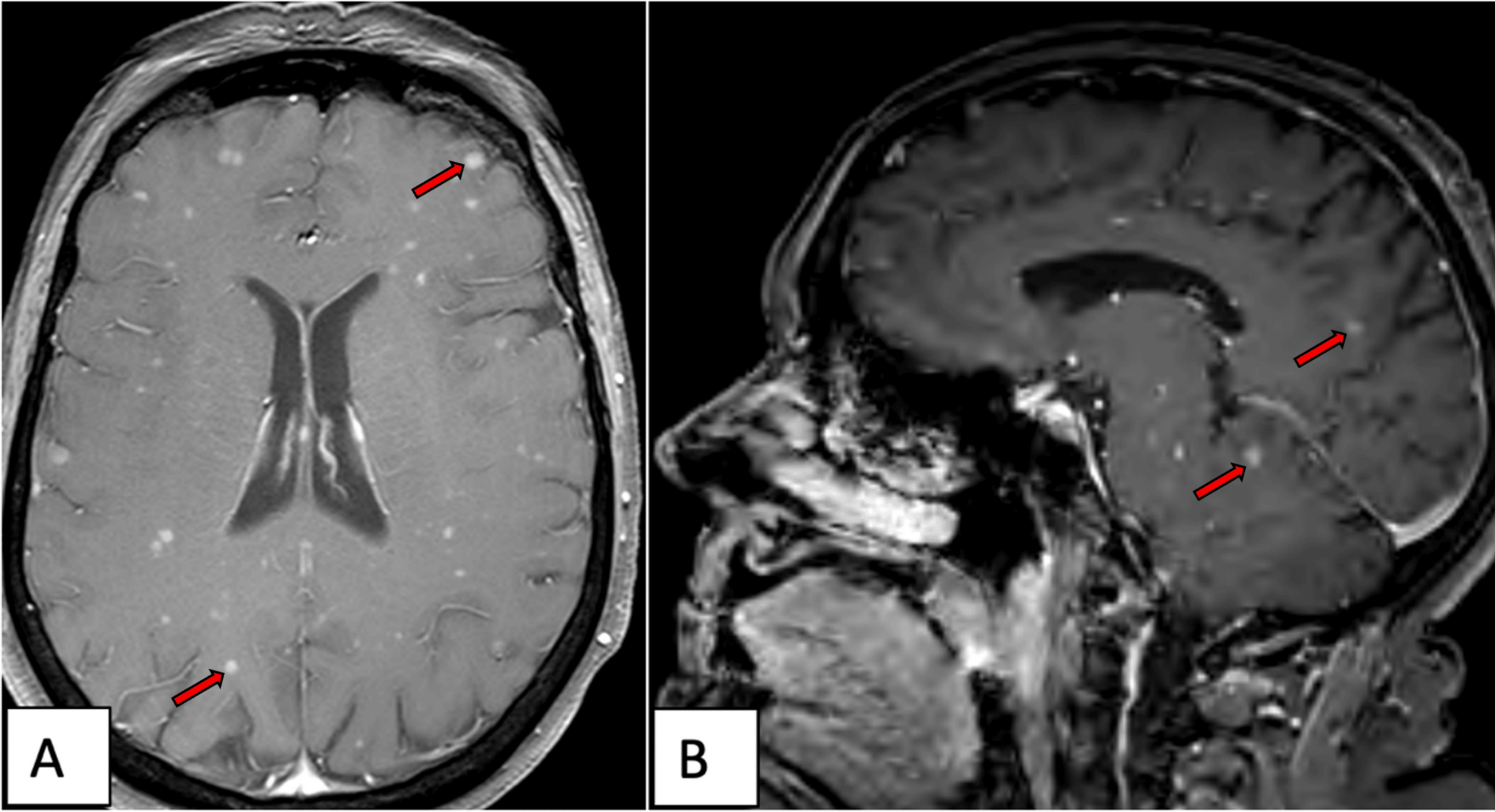
Brain MRI detailing widespread supratentorial and infratentorial atypical carcinoid metastases (red arrows) that progressed through systemic therapy. A) Axial and B) sagittal views

Given her high functional status and the asymptomatic nature of the lesions, treatment with maintenance capecitabine and temozolomide was continued. The patient continued to receive systemic therapy and undergo repeat brain imaging for another two years, until she began to develop headaches and dizziness requiring dexamethasone (4 mg daily). Repeat MRI brain showed an increase in the number of punctate lesions throughout the brain and brainstem, with no clear leptomeningeal involvement (Figure 2). Of note, no lesions were found to be within five millimeters of the hippocampi bilaterally, making her a viable candidate for HS-WBRT as per Radiation Therapy Oncology Group (RTOG) 0933 [8].

**Figure 3:**
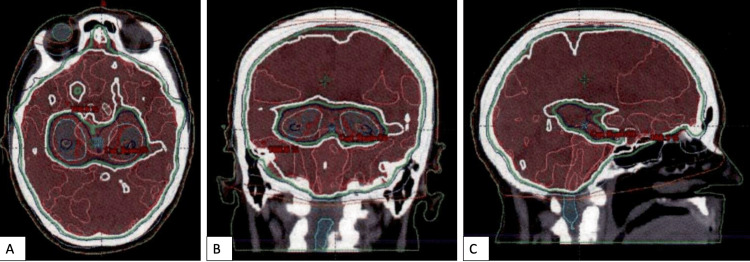
HS-WBRT plan showing the planning target volume (red) covered with the prescribed 30 Gy isodose line (white) A) Axial, B) coronal, and C) sagittal views. HS-WBRT: Hippocampal-sparing whole brain radiotherapy

A HS-WBRT plan was generated, with planning target volume (PTV) prescribed to the 100% isodose line (Figure 3). The patient was prescribed 30 Gy in 10 fractions using daily image-guided 6X VMAT photon therapy (600 MU/min). The dose was optimized and calculated with the AcurosXB planning system and delivered on Truebeam using two coplanar arcs (Varian Medical Systems, Palo Alto, US). PTV coverage was met, with at least 90% of the PTV receiving 30 Gy (goal >30Gy), and at least 98% of the PTV receiving 25.5 Gy (goal >25Gy). All dose constraints were respected, including the maximum dose to cover 100% of the hippocampus (D100) being 7.5 Gy (goal ≤9Gy) and the maximum dose (Dmax) delivered to the hippocampus being 14.5 Gy (goal≤16Gy).

**Figure 4:**
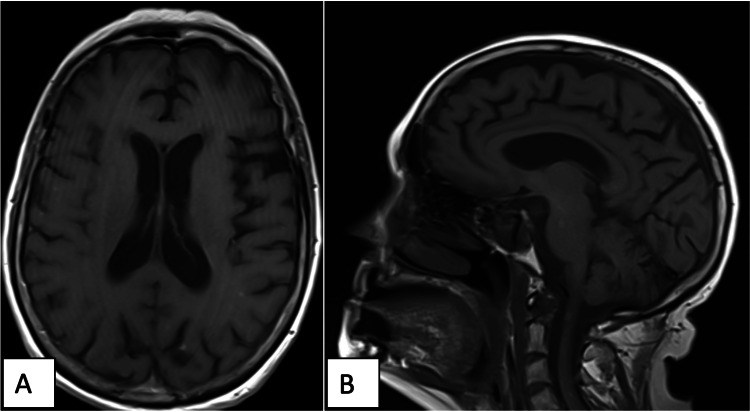
Brain MRI at 11 months after HS-WBRT showing significantly decreased size and number of metastases A) Axial and B) sagittal views. HS-WBRT: Hippocampal-sparing whole brain radiotherapy.

The patient tolerated the treatment well and experienced only grade one fatigue by the end of her radiation course. At her one-month follow-up, she denied any acute toxicities, had discontinued dexamethasone, and exhibited ECOG-PS of zero. Genetic testing revealed that her tumor had mutations in epidermal growth factor receptor (EGFR) and the platelet-derived growth factor receptor β (PDGF-β). As per the multi-disciplinary tumor board recommendations, she was placed on everolimus, but she discontinued the medication four months post-radiation due to poor tolerance and declined further systemic therapy. Due to the metastases in her upper cervical and thoracic spine, the patient developed progressive bilateral lower extremity weakness requiring a wheelchair for mobility. These symptoms prompted her to move into a nursing home six months after receiving HS-WBRT, which limited her follow-up. When examined 10 months after the HS-WBRT, the patient exhibited ECOG PS of two, with bilateral lower extremity strength rated two out of five throughout. However, she remained oriented to person, place, and time, was able to answer questions appropriately, and denied lapses in her memory or cognition. Repeat MRI brain at 11 months post-radiation revealed decreased as well as stable size and number of brain metastases, with no new ones identified, demonstrating effective local control (Figure 4). The patient’s systemic disease unfortunately continued to progress elsewhere and she passed away 13 months after delivery of HS-WBRT.

## Discussion

Carcinoids are rare neuroendocrine tumors (NETs) that arise from enterochromaffin cells, characterized by their ability to stain with chromium or chrome salts due to the presence of 5-HT [9]. Diagnosis is based upon histology, with positive immunochemical staining for one or more neuroendocrine markers (chromogranin A or synaptophysin) or electron microscopy in which the tumor cells are found to contain membrane-bound secretory granules containing biogenic amines, peptides, or tachykinins [9]. Based on their histological patterns, carcinoid tumors are characterized as typical or atypical [3]. Typical carcinoids display a well-differentiated histologic pattern and are classified as insular, trabecular, glandular, mixed, or undifferentiated. Atypical carcinoids, however, display increased nuclear atypia, focal necrosis, or high mitotic indices. Histopathologic and clinical diagnosis of atypical carcinoma requires neuroendocrine morphology and either coagulative necrosis or mitotic counts of 2-10/2 mm^2 ^of viable tumor [10].

In addition to the differentiation and proliferation rate, the presence of metastases is known to be a major prognostic factor for NETs [11,12]. Brain-metastatic NETs have been frequently reported, but the standards for detailed treatment and outcomes for these metastases have not been fully established. Furthermore, the roles of stereotactic radiosurgery (SRS) or WBRT in the optimal management of brain metastases remains under debate, particularly for sparing cognition [11,13-20]. SRS combined with WBRT has generally been recommended for patients with radiographically-evident metastases from small cell lung cancer (SCLC) [11,12,16]. The American Society of Clinical Oncology (ASCO), Society for Neuro-Oncology (SNO) and American Society for Radiation Oncology (ASTRO) guidelines have indicated that SRS alone, as opposed to WBRT or a combination of WBRT and SRS, is considered suitable therapy for patients with oligometastatic disease with the exception of neuroendocrine tumors such as SCLC, since these histologies were excluded from key randomized trials [20]. For patients with SCLC but no radiographically-evident brain metastases, prophylactic cranial irradiation has been shown to confer benefits on survival and reduced incidence of brain metastases [17]. Whether such benefits would be conferred to bronchogenic NETs has not yet been clarified.

Prior limited data has demonstrated a trend towards improved survival in patients who undergo surgery followed by adjuvant WBRT [7,10]. However, the majority of patients experience cognitive decline after WBRT, raising concerns about its toxicity. The results from the multi-institutional phase III National Surgical Adjuvant Breast and Bowel Project, Radiation Therapy Oncology Group, and Gynecologic Oncology Group (NRG) Oncology CC001 trial comparing non-HS-WBRT plus memantine against HS-WBRT plus memantine demonstrated how conformal hippocampal avoidance preserves cognitive function without significant additional toxicity [13,14]. Studies have shown the use of temozolomide, either as monotherapy or in combination with capecitabine in lung NETs [12,18]. Temozolomide’s activity against NETs and its ability to penetrate the blood-brain barrier makes it useful in treating and managing radiographically and clinically evident brain-metastatic carcinoids without posing the same degree of risk for neurocognitive decline as traditional WBRT. This is in keeping with current guidelines, which recommend cytotoxic chemotherapy for chemosensitive tumors in patients with asymptomatic or small brain metastases [19]. Due to the poor prognosis and rarity of non-operative, brain-metastatic atypical carcinoids, data for management and follow-up is limited. Current guidelines recommend follow-up with appropriate CT chest/abdomen/pelvis imaging and blood/urine markers three to six months after curative resection, then every six to 12 months for at least seven years for stage II-III disease [19]. In addition to these recommendations, we propose that patients may benefit from undergoing regular brain imaging in three- to six-month intervals if they present with more advanced stages of disease (i.e. stage IIB or greater) or show disease progression on surveillance imaging. This may confer earlier diagnosis of intracranial metastases and prompt necessary changes in the systemic therapy. Although follow-up with this patient was limited by the aggressive nature of her systemic disease, her case provides insights into the safety and efficacy of HS-WBRT for these kinds of presentations, as well as the challenges of achieving effective local and systemic control against atypical carcinoids through multiple lines of therapy.

## Conclusions

Brain-metastatic atypical carcinoid is a rare occurrence that confers a poor prognosis. This case details successive palliation of intracranial metastases using HS-WBRT, showing its utility in conferring excellent local control while preserving cognition. However, this case also testifies to the aggressive nature of atypical carcinoids, warranting active surveillance and treatment. More frequent surveillance for intracranial metastases in patients with atypical histologies, advanced stage on initial presentation, or signs of clinical or radiographic disease progression may prompt earlier diagnosis, leading to necessary changes in systemic therapy and earlier inclusion of brain-directed radiotherapy. Multidisciplinary approach and treatment is needed from diagnosis onwards. Well-designed prospective randomized trials and meta-analyses focused on atypical pulmonary carcinoids are warranted.
